# Symptom Profiles and Anatomical Distribution of Deep Infiltrating Endometriosis

**DOI:** 10.7759/cureus.103702

**Published:** 2026-02-16

**Authors:** Varna Jammula, Lucas Lee, Jenny Huynh, Mandy Zhu, Chandra Spring-Robinson, Matthew Schultzel, Sherli Koshy-Chenthittayil

**Affiliations:** 1 College of Osteopathic Medicine, Touro University Nevada, Henderson, USA; 2 General and Colorectal Surgery, United Medical Doctors - La Jolla, San Diego, USA; 3 Obstetrics and Gynecology, United Medical Doctors - La Jolla, San Diego, USA; 4 Research, Touro University Nevada, Henderson, USA

**Keywords:** abdominal pain, colorectal surgery, dysmenorrhea, general surgery, gynecology

## Abstract

Introduction: Endometriosis is known to affect women worldwide, and most commonly involves chronic pelvic pain and infertility. Deep infiltrating endometriosis (DIE) is a condition that may involve the bladder, ureter, and bowel. In our study, we evaluated symptoms and locations of endometrial lesions in patients with DIE to improve efficiency in management.

Methods: Data were collected retrospectively from electronic medical records (Epic EHR; Epic Systems, Verona, USA) at the United Medical Doctors General and Colorectal Surgery Clinic in La Jolla, San Diego, California, USA. We analyzed 22 patients with an average age at diagnosis of 33.6 years. We utilized frequency distribution and heat maps to summarize endometriosis lesion locations.

Results: In our sample of 22 patients, the most common site of endometrial lesions was the bowel, and only three patients had a positive colonoscopy. Lesion burden was found to be the most significant in the pelvic peritoneum/ligaments. Abdominal pain was reported to be the most common symptom across multiple anatomical sites, followed by dyschezia, constipation, and painful menstrual cramps. Additionally, gastrointestinal symptoms, including nausea, diarrhea, and pain with bowel movements, occurred most frequently in patients with bowel, pelvic peritoneum, and ovarian lesions.

Conclusion: The most common symptoms observed in this study are also seen in many gastrointestinal disorders, highlighting the importance of considering DIE as a potential diagnosis in patients with bowel symptoms. Future research should focus on prospective multicenter studies to validate imaging protocols for DIE and to better differentiate related gynecological and gastrointestinal symptomology.

## Introduction

Endometriosis is a highly prevalent condition, affecting over 10% of women [1]. It is commonly associated with the clinical presentation of infertility and chronic pelvic pain, as well as other symptoms such as dysmenorrhea, dyspareunia, and dyschezia [2]. Endometrial lesions may involve the ovaries, uterine ligaments, pouch of Douglas, and fallopian tubes. Additional endometrial implants can be found in extrapelvic locations such as the gastrointestinal tract, lungs, diaphragm, abdomen, and pericardium [3]. The most widely accepted theory for the pathogenesis of endometriosis involves retrograde menstruation, where reflux of blood from the fallopian tubes to the peritoneum during menstruation allows cells to implant in the peritoneal cavity. Endometrial lesions found in the pouch of Douglas and uterosacral ligaments are hypothesized to result from atypical migration and differentiation of Müllerian duct remnants [4].

There are three recognized forms of endometriosis within the pelvic region: deep infiltrating endometriosis (DIE), peritoneal endometriosis, and ovarian endometrial cysts [5]. DIE is defined as infiltration of more than 5 mm below the peritoneum, which may involve the bladder, ureter, and bowel [4]. It most commonly involves the uterosacral ligaments, the rectovaginal space, the upper third of the posterior vaginal wall, the bowel, and the urinary tract [6]. The prevalence of DIE is 1-2%, and about 95% of cases are associated with severe pain [7]. Although less frequent in occurrence in comparison to other categories of endometriosis, DIE is associated with a higher risk of infertility and more severe cases of dysmenorrhea, making it a major case of concern for efficiency in management, treatment, and safety for pregnancy and delivery outcomes [8]. While clinical symptoms and imaging may aid in the preliminary diagnosis of endometriosis, the gold standard is laparoscopy with histopathological examination. Surgical treatment of endometriosis has a high success rate, with 50-80% symptom reduction [9]. The purpose of this study was to evaluate the symptoms and location of endometrial lesions in patients with DIE. The secondary objective was to examine colonoscopy findings in relation to lesion location.

## Materials and methods

The de-identified data were collected from the electronic medical records (Epic EHR; Epic Systems, Verona, USA) of patients at the United Medical Doctors General and Colorectal Surgery Clinic in La Jolla, San Diego, California, USA, by a medical assistant. Patients were included in the study if they had a diagnosis of endometriosis, as determined by histopathological examination, and a surgical treatment of endometriosis involving bowel between 2020 and 2025. The following information was collected from the records: year of birth, age at diagnosis, ethnicity, BMI at time of diagnosis, onset of diagnosis, symptoms, location of endometriosis (based on imaging, scope, or intraoperatively), whether the colonoscopy was positive for endometriosis, type of surgical procedure, date of surgery, and age at time of surgery. Statistical analyses and figure generation were performed using R version 4.5.1 (R Foundation for Statistical Computing, Vienna, Austria). The following R packages were used: ggplot2, dplyr, tidyr, forcats, stringr, scales, e1071, readr, janitor, and grid.

## Results

The sample size included 22 patients, and the average age at diagnosis of endometriosis was 33.6 years (Table 1). There were three patients with missing data regarding age at diagnosis and 12 patients with missing data regarding BMI at diagnosis. Ethnicity was reported by seven patients (five White patients and two Hispanic patients), while 14 declined to disclose their ethnicity and one had missing data.

**Table 1:** Average age and BMI of patients at diagnosis

Demographic	Mean ± SD	Sample size
Age at diagnosis	33.6 ± 8.5	19 of 22
BMI at diagnosis	25.8 ± 7.2	10 of 22

**Figure 1:**
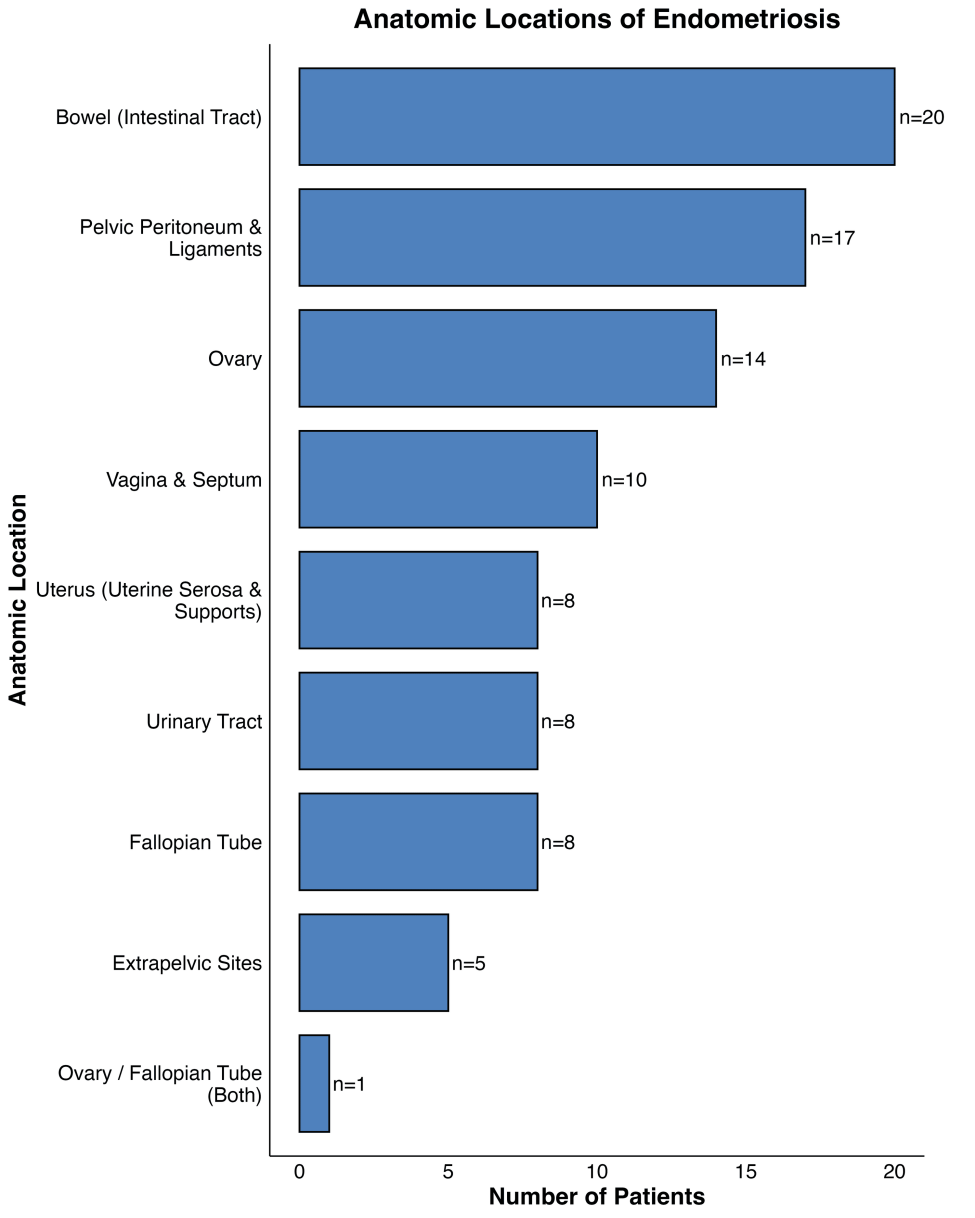
Anatomic locations of the endometriosis

The most frequent site of endometriosis (Figure 1) in this cohort was the bowel, affected in 20 patients. The pelvic peritoneum and ligaments were the second most common site, affected in 17 patients, followed by the ovary, which was involved in 14 patients. Other locations included the vagina and septum (10 patients), uterus (uterine serosa and supports) (eight patients), urinary tract (eight patients), and fallopian tube (eight patients). Less frequent locations were extrapelvic sites (five patients) and combined ovary/fallopian tube involvement (one patient). Additionally, two patients were categorized as having a disease stage without a specific location, and one patient had missing data.

**Figure 2:**
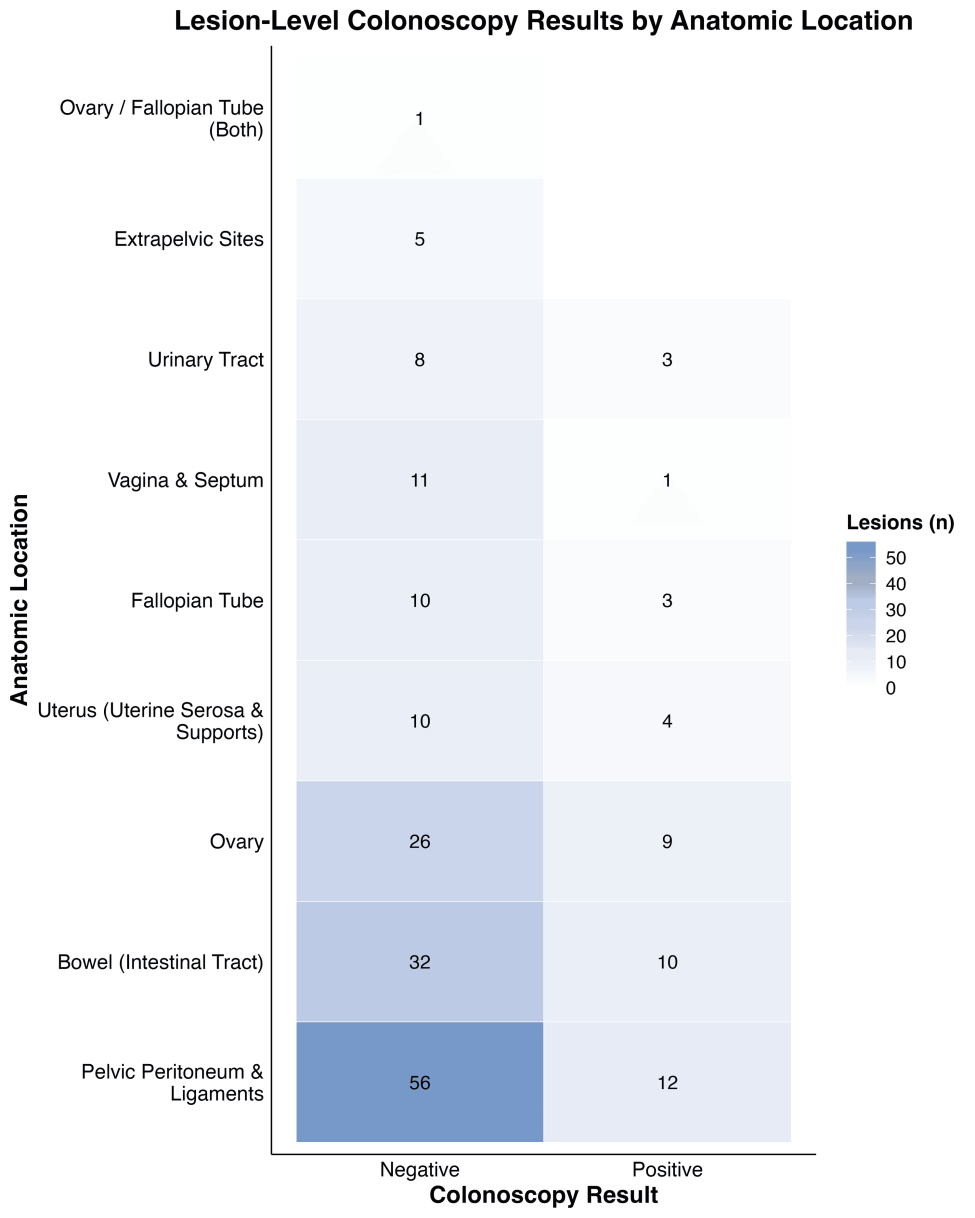
Heat map showing lesion locations in relation to colonoscopy results

When considering lesion burden (Figure 2), the pelvic peritoneum and ligaments accounted for the largest proportion of lesions, with a total of 68 (56 colonoscopy-negative and 12 colonoscopy-positive). The bowel was the second most common site, with 42 lesions (32 negative and 10 positive), followed by the ovary with 35 lesions (26 negative and nine positive). Smaller numbers of lesions were observed in the uterus, fallopian tube, vagina and septum, and urinary tract. A positive colonoscopy for endometriosis did not correspond proportionally to lesion burden, suggesting limited sensitivity of colonoscopy for detecting endometriosis.

**Figure 3:**
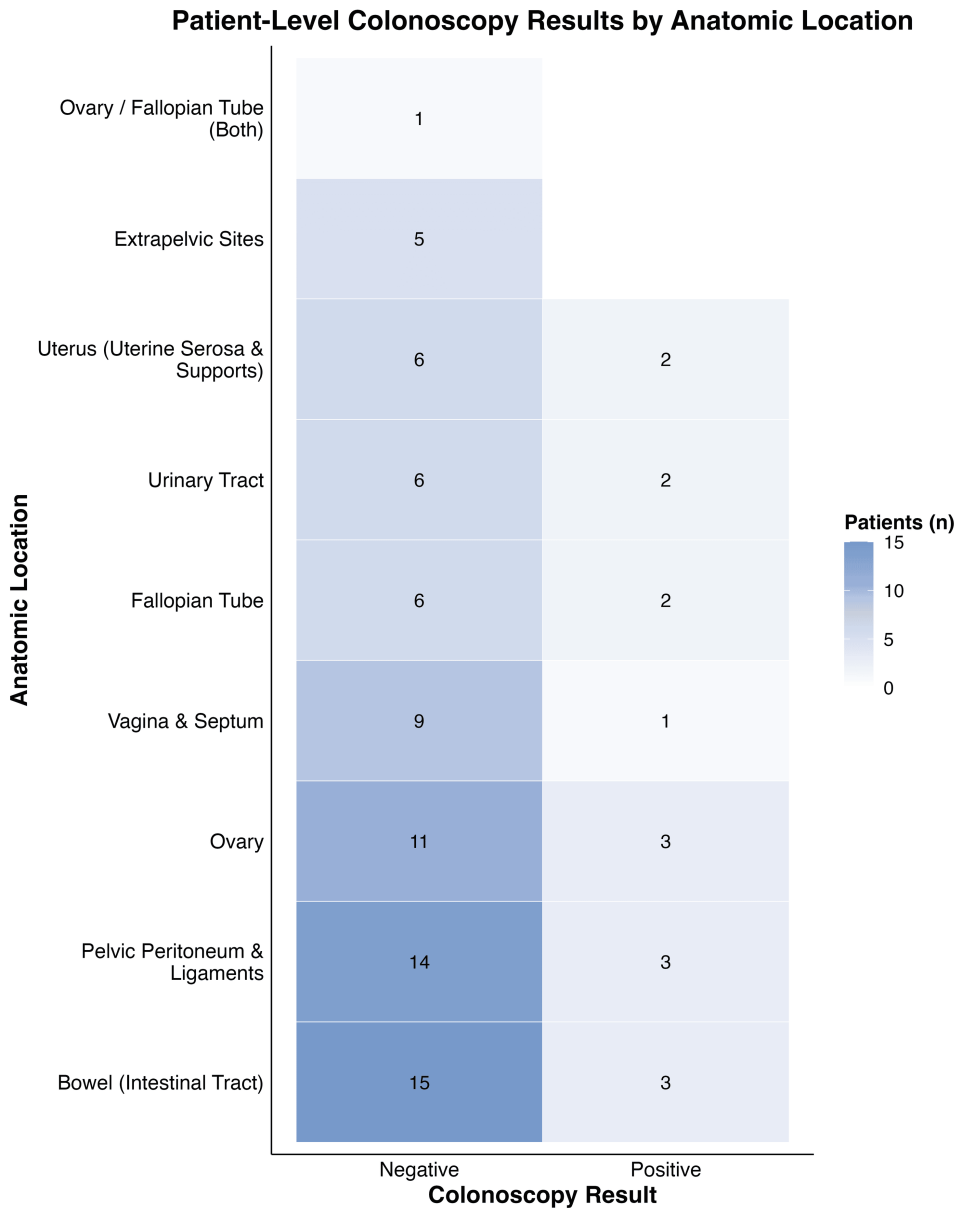
Heat map showing the number of patients by lesion location and corresponding colonoscopy results

It was observed that the bowel was the most frequently involved site (Figure 3), affecting 18 patients in total (15 colonoscopy-negative and three colonoscopy-positive). The pelvic peritoneum and ligaments were the next most common, affecting 17 patients (14 negative and three positive). Ovarian involvement was also frequent (14 patients; 11 negative and three positive), followed by the vagina and septum (10 patients; nine negative and one positive). A smaller number of patients had involvement of the uterus, fallopian tube, urinary tract, or extrapelvic sites. The number of positive colonoscopies for endometriosis was relatively low compared with the overall patient burden, again suggesting limited sensitivity of colonoscopy for detecting endometriosis across sites.

**Figure 4:**
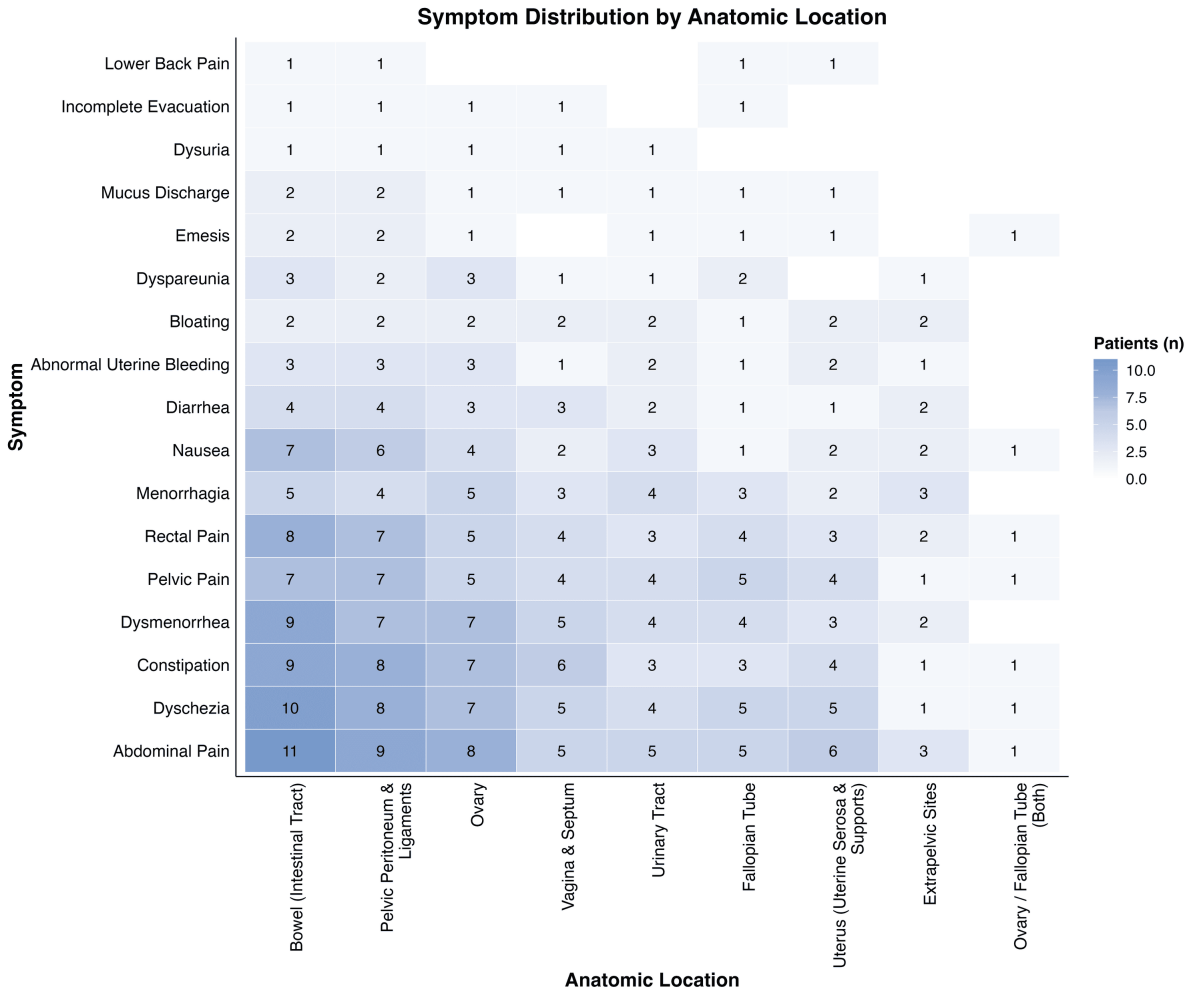
Heat map showing the number of patients with varying symptoms across anatomic locations of lesions

Abdominal pain was the most frequently reported symptom, occurring across nearly all anatomic sites, with the highest co-occurrence in the bowel, pelvic peritoneum and ligaments, and ovary, as shown in Figure 4. Other common symptoms included dyschezia, constipation, painful menstrual cramps, pelvic pain, and rectal pain, each reported across multiple lesion sites. Rectal pain and rectal bleeding were most closely associated with bowel and pelvic peritoneum involvement. Gynecologic symptoms such as painful menstrual cramps, heavy menstrual bleeding, and pain during intercourse were broadly distributed across the ovary, uterus, and pelvic peritoneum. Gastrointestinal symptoms, including nausea, diarrhea, pain with bowel movements, and bloating, were most frequent in patients with bowel, pelvic peritoneum, and ovarian lesions. Less frequently reported symptoms, such as emesis, incomplete evacuation, and lower back pain, were not strongly associated with a specific lesion site. Overall, symptom expression overlapped across multiple anatomical sites, underscoring the heterogeneity of clinical presentation.

**Figure 5:**
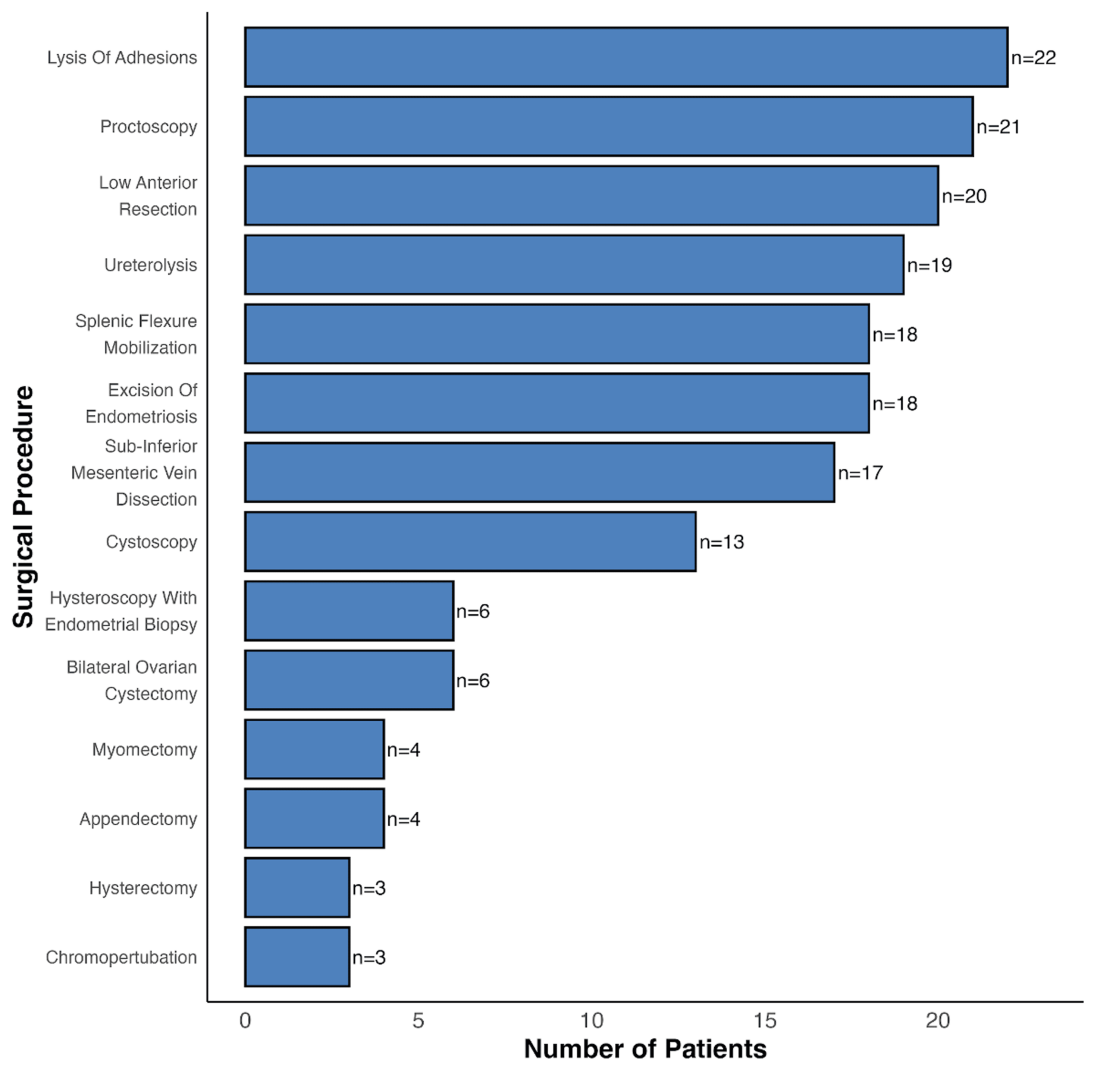
Frequency chart of surgical procedures among patients

Across patients, the most common procedures (Figure 5) were lysis of adhesions (22 patients), intraoperative proctoscopy (21 patients), and low anterior resection (20 patients). Hysterectomy and chromopertubation were among the least common procedures, with only three patients undergoing each.

## Discussion

The present study investigated the symptom profiles and anatomical distribution of DIE in patients at a general and colorectal surgery clinic. The most common sites of lesions were the bowel, followed by the pelvic peritoneum and ligaments. The largest proportion of lesions was located in the pelvic peritoneum and ligaments, followed by the bowel. The most common symptom was abdominal pain, which frequently co-occurred with bowel endometriosis. Dyschezia, constipation, and dysmenorrhea were also common in these patients. These symptoms also co-occurred with lesions located in the pelvic peritoneum, peritoneal ligaments, and ovary.

Interestingly, while there were 42 lesions identified in the bowel during surgery, only 10 cases were positive for endometriosis on colonoscopy. This may be explained by the endometrial lesions occurring on the serosa rather than infiltrating into the mucosa, making them difficult to detect during colonoscopy. Several studies have also identified the poor sensitivity of colonoscopy for detecting bowel endometriosis [10-15]. In one study, the authors reported a sensitivity of 7% and a specificity of 98% for colonoscopy in the detection of intestinal endometriosis [10]. Other studies have noted that biopsy during colonoscopy may confirm the diagnosis of bowel endometriosis, but this remains useful only when there is visible mucosal involvement [11,12]. Given the low sensitivity and invasive nature of colonoscopy, it should be reserved for confirming a suspected diagnosis of bowel endometriosis. While some studies have compared alternative methods of diagnosis, such as ultrasound and MRI [13,14], the gold standard for diagnosing endometriosis remains laparoscopy with pathological examination [15].

Many of the symptoms experienced by the patients in this study are commonly reported among those diagnosed with irritable bowel syndrome (IBS) or other gastrointestinal diseases. These nonspecific symptoms, such as abdominal pain, bloating, dyschezia, and constipation, overlap with a range of gastrointestinal disorders such as celiac disease and colorectal cancer, further complicating the diagnostic process of endometriosis [16-18]. The overlapping symptoms of endometriosis and gastrointestinal symptoms that mimic IBS have been studied. Due to the lack of distinguishing features in the symptom profiles of these patients, they may be subjected to extensive gastrointestinal workup before endometriosis is considered, resulting in diagnostic delays and increased healthcare costs [18,19]. The cyclical nature of endometriosis symptoms may often be overlooked in gastrointestinal workups, further causing a diagnosis of endometriosis to be missed. Therefore, it is important to consider DIE as a potential diagnosis in patients with bowel symptoms.

This study involved a small sample size and had limited demographic information on the patients included. The patients were all evaluated at a general and colorectal surgery clinic after an initial visit to their gynecologist, potentially introducing a selection bias in this sample. The lack of a standardized symptom assessment tool may also affect the generalizability and reproducibility of the findings. Nevertheless, the present study described the most common lesion locations and symptom profiles of DIE patients, which are important for clinicians to consider in the complex evaluation and management of DIE. Future research should focus on prospective multicenter studies to validate imaging protocols for DIE. Studies should also explore the role of collaboration between gastroenterology and gynecology to evaluate and manage patients with overlapping gastrointestinal and gynecologic symptoms. Additionally, studies on symptom improvement and pregnancy rates after resection of DIE may provide further information on the benefits of invasive diagnostics and treatment methods.

## Conclusions

This study highlights DIE as a disease commonly involving the bowel and pelvic peritoneum and associated with nonspecific gastrointestinal symptoms. These symptoms frequently overlap with disorders such as IBS, contributing to diagnostic challenges and delays. The poor sensitivity of colonoscopy for detecting bowel endometriosis reinforces existing evidence indicating that mucosal evaluation alone is insufficient and laparoscopy with histopathologic examination remains the diagnostic gold standard. Despite limitations including small sample size, possible selection bias, and the lack of standardized symptom assessment, these findings remain clinically relevant. They underscore the importance of maintaining a high index of suspicion for DIE in patients presenting with bowel symptoms and support the need for multidisciplinary collaboration and further research to optimize diagnostic strategies and management.
